# Structural and Managerial Risk Factors for COVID-19 Occurrence in French Nursing Homes

**DOI:** 10.34172/ijhpm.2022.6741

**Published:** 2022-03-06

**Authors:** Aline Corvol, Kevin Charras, Joaquim Prud'homm, Fabien Lemoine, Fabien Ory, Jean François Viel, Dominique Somme

**Affiliations:** ^1^CIC 1414, Inserm, CHU Rennes, Univ Rennes, Rennes, France.; ^2^ARENES, UMR 6051, ARENES, CHU Rennes, Univ Rennes, Rennes, France.; ^3^Department of Geriatrics, CHU Rennes, Rennes, France.; ^4^Living Lab Ageing and Vulnerability, CHU Rennes, Rennes, France.; ^5^Inserm, LTSI, UMR 1099, Univ Rennes, Rennes, France.; ^6^Department of Epidemiology and Public Health, CHU Rennes, Univ Rennes, Rennes, France.

**Keywords:** COVID-19, Nursing Home, Lockdown, Communal Dining, Personal Protective Equipment, France

## Abstract

**Background:** Nursing home (NH) residents accounted for half of the deaths during the 2020 spring wave of the coronavirus disease 2019 (COVID-19) epidemic in France. Our objective was to identify structural and managerial factors associated with COVID-19 outbreaks in NHs.

**Methods:** We conducted in July 2020 a retrospective study by questionnaire addressed to NH directors in the Brittany region of France. The questions related to structural characteristics of the establishment, human resources, and crisis management decisions. The primary endpoint was the occurrence of at least one confirmed case of COVID-19 among residents between March 1, 2020 and May 31, 2020. The secondary endpoint was total mortality during this period. We used multivariate regressions to identify factors associated with these outcomes.

**Results:** Responses were collected from 231 NHs hosting 20,881 residents, representing a participation rate of 47%. In 24 (10%) NHs, at least one resident presented confirmed COVID-19. NHs often implemented stringent protective measures, with 65% of them choosing to confine residents to their rooms. In multivariate analysis, factors associated with a reduced risk of case occurrence were in-room meal service, early ban of family visits, and daily access to an outdoor space. No association was found between mortality and the factors studied. Our results show an early and strict implementation of lockdown measures, with good epidemiological results in a context of shortage of personal protective equipment (PPE) and non-vaccination. Nevertheless, it raises ethical questions concerning respect of residents’ wellbeing and rights.

**Conclusion:** Cessation of communal dining seems to be the main measure likely to be effective in preventive terms. It does not seem that room lockdown and cessation of group activities should be recommended, particularly if mask wearing is possible.

## Background

**Key Messages**

** Implications for policy makers**

- In-room meal service, daily access to outdoor spaces, and an early ban on family visits were associated with reduced coronavirus disease 2019 (COVID-19) epidemic risk in nursing home (NH), in a context of limited infection rate in general population.
- Our results do not support preventive room lockdown and cessation of group activities, despite the context of shortage of personal protective equipment (PPE) and non-vaccination.
- No association was found between size, location or human resources of NHs, and outbreak risk.

** Implications for the public**

Nursing home (NH) residents accounted for half of the deaths during the 2020 spring wave of the coronavirus disease 2019 (COVID-19) epidemic in France. We conducted in July 2020 a retrospective study by questionnaire addressed to NH directors in the Brittany region of France. Responses, collected from 231 NHs hosting 20 881 residents, showed that NHs often implemented stringent protective measures, with 65% of them choosing to confine residents to their rooms. Factors associated with a reduced risk of case occurrence were in-room meal service, early ban of family visits, and daily access to an outdoor space. Cessation of communal dining seems to be the main measure likely to be effective in preventive terms. It does not seem that room lockdown and cessation of group activities should be recommended, particularly if mask wearing is possible.

The coronavirus disease 2019 (COVID-19) pandemic affected France in the first half of 2020, with a first wave between March and May.^1,2^ The French government responded by imposing a strict national lockdown from 17 March to 11 May (closure of schools and non-essential shops).^3^ Nursing homes (NHs), in France as elsewhere, were particularly affected by the epidemic^2,4^: among 29 021 deaths during the first wave, 14 178 concerned residents of long-term care facilities.^5^ These figures are likely underestimated, notably because of the absence of systematic reverse transcription polymerase chain reaction (RT-PCR) testing in NHs at the start of the epidemic.^6^ Our study was in Brittany, a region of western France that was relatively little affected by the first wave of COVID-19,^4^ despite a first cluster from March 1, 2020,^2^ with a total of 2890 confirmed cases among 3 340 400 inhabitants between March and May.^7^

In France, NHs (ie, établissement d’hébergement pour personnes âgées dépendantes) are long-term care facilities, which house 10% of people aged over 75.^8^ Over half the residents have a severe loss of functional independence, 8 out of 10 have cognitive disorders, and median survival is 28 months.^9^ These public, private for-profit or private not-for-profit homes provide social and nursing support to residents. A coordinating physician deals with organizational matters. However, residents are mainly cared for by general practitioners based outside. There is usually no nurse overnight.

Lockdown measures in French NHs were initiated on 11 March, with a governmental recommendation to ban all non-professional visits.^10^ Mask wearing at that time was officially advised against, except in hospital sectors receiving people infected by COVID-19, because of the risk of a break in supply.^11^ On 28 March, the government recommended a strengthening of social distancing measures in NHs, including going as far as confining residents to their rooms.^12^ It was only on 31 March that the Public Health Council recommended systematic mask wearing by NH personnel and the ending of communal meals and activities.^13^ Finally, on 20 April, a governmental protocol authorized visits under strict supervision (dedicated secure space, surveillance by a caregiver to ensure respect of social distancing).^14^

Faced with the twofold necessity to prevent an outbreak while maintaining an acceptable quality of life for the residents, NHs responded according to their structural characteristics, the availability of personal protective equipment (PPE) and the local dynamics of the epidemic.^15^ These choices were made under uncertainty,^16^ heightened by difficulties of access to RT-PCR tests during the first wave of the epidemic. Until mid-April, RT-PCR tests were mainly performed by mobile teams that only intervened when there was a cluster of at least two suspected cases among residents.^17^ When one of the tests was positive, all symptomatic residents of the same NH were considered “probable cases,” without a new test being performed. Symptoms have been defined in this study as “fever, respiratory signs or sudden fatigue.” There was no systematic testing of residents or of caregivers.

Our study had a quantitative component involving administration of a questionnaire, the results of which are presented here, and a qualitative component. The primary objective was to analyze retrospectively the factors associated with the occurrence of at least one confirmed case in NHs. Our hypotheses related to the effect of the different protective measures, the search for a link between the risk of an outbreak and the human resources, the size and location of the NH. The secondary objective was to analyze the factors associated with the number of deaths among residents during this period. This variable is important in taking into account the proportion of deaths linked to COVID-19 for which no diagnosis was made, and the potentially harmful effects of lockdown measures on health in general.^18^ Lastly, the study allowed us to document practices concerning the lockdown of residents and the main difficulties identified by the management teams.

## Methods

###  Setting and Partners

The research team included geriatricians directly involved in providing support to NHs,^17^ a physician-epidemiologist, an advanced practice nurse and a psychologist. The questionnaire was drawn up and administered in partnership with the regional support centre for quality of care,^19^ the Support Centre for the Prevention of Nosocomial Infections, and the National Association of Coordinating Physicians for NHs.

###  Participants

 We included all NHs in the Brittany region of France, using the list of the regional support centre for quality of care. The support centre first contact them to ensure they were not opposed to participation. The questionnaire was then emailed to them on June 23, 2020, with three follow-up reminders in July.

###  Endpoints

 The primary endpoint was the occurrence of at least one case of COVID-19 among residents between March 1, 2020 and May 31, 2020, confirmed by a positive RT-PCR test using a nasopharyngeal swab or by a chest scan. The secondary endpoint was the total number of residents who died during this period.

###  Variables Observed

 The research team designed a questionnaire using literature data and governmental recommendations. Particular effort was made to keep it concise. It was revised and amended by all partners and then tested by three NH directors who estimated that 20 to 30 minutes were needed to complete the questionnaire.

 The questionnaire comprised 35 multiple-choice questions on the NH structural characteristics and human resources, lockdown measures implemented before the occurrence of a case, difficulties experienced concerning observance of the protective measures (4-point Likert scale: very easy, rather easy, sometimes difficult, very difficult), and the consequences of the outbreak. The questionnaire ended with two open questions concerning resources that were in short supply during crisis management and the most useful resources.

More precisely, the explanatory variables collected concerned the location of the NH (rural or urban, distinguishing between city centres, isolated towns, and suburbs; the administrative department); the number of residents and their average functional dependence^21^; the proportion of double rooms; human resources such as the presence of a coordinating physician or a hygienist, the ratios of nurses, healthcare assistants, and all salaried personnel per resident. Questions also related to lockdown measures implemented before the occurrence of a first case: number of residents per sector, residents confined to their rooms, daily access to an outside area, in-room meal service, cessation of group activities. The last questions related to possible misuse of masks and gowns (for example, use for longer than recommended by the manufacturer, re-use of disposable materials, use of unlicensed or home-made equipment), the pre-emptive or delayed implementation of recommendations, concerning a ban on visits^10^ and systematic mask wearing by professionals.^3^

###  Statistical Analysis

In a multivariate logistic regression, the statistical unit was the NH and the variable explained the presence or absence of at least one case of COVID1-19 among residents during the period considered. All the explanatory variables mentioned above were considered, the number of residents being a forced variable. The territorial subdivision (four in the Brittany region) in which the NH was located was forced in the multivariate model to account for variation in epidemic dynamics across Brittany. All other explanatory variables with a degree of significance <.20 in univariate analyses were included in the multivariate model.^20^ The results are expressed as odds ratios (ORs) with their 95% confidence intervals (CIs). A quasi-Poisson regression was then used to explain the number of all-cause deaths in the NH. We introduced the number of residents as an offset variable. The same explanatory variables were studied and the same strategy for selection of explanatory variables was followed. The results are expressed in the form of relative risk with its 95% CI. All statistical analyses were done using R software (version 4.0.2).

## Results

###  Description of the Sample

 Of the 492 NHs identified, 2 refused to receive the questionnaire. We included all respondents in the analysis. The 231 NHs that completed the questionnaire (participation rate 47%) accommodated 20 881 residents. Of these 231, 66% were public, 30% private not-for-profit, and 4% for-profit. Most (56%) had no double rooms. Each of the 231 NHs accommodated an average of 90 residents (range 13 to 448; median 76). The position of coordinating physician was not filled in 35% of them and more than 5 different general practitioners attended to the residents in 71%. Almost half (48%) of the NHs had a professional whose time was in part devoted to hygiene, and 58% organized hygiene training during this period. There were 0.07 full-time equivalent nurses and 0.29 healthcare assistants per resident.

###  Morbidity-Mortality

 In all, 1307 deaths were recorded among the 20 881 residents of the NHs questioned during these 3 months, ie, a death rate of 6%. In 24 (10%) NHs, at least one resident presented a confirmed COVID-19 infection. The total number of confirmed or probable cases was 241 (9% of the 2567 residents hosted in those 24 NHs), ranging from 1 (9 NHs) to 45, with an average of 9 cases/NH. Sixty-four deaths were associated with COVID-19, ie, a death rate of 26% (64/241) for probable or confirmed cases. Overall, COVID-19 was associated with 5% of the deaths that occurred during this period in the 231 NHs.

 Forty-two NHs (18%) reported that at least one staff member had tested positive, the total being 149 employees, symptomatic or not. Systematic testing of staff was carried out in 39 NHs (17%).

###  Outbreak Management 

 Our results show that NHs frequently implemented lockdown measures in advance of governmental recommendations. Thus, 92% of the NHs asked all caregivers to wear a mask before the recommendation of 31 March, and 31% of them from the first fortnight of March. Likewise, 51% of NHs banned family visits before the governmental recommendation was issued on March 11, 2019. Overall, 65% of the NHs chose to confine residents to their rooms, 80% served meals in the residents’ rooms and 62% stopped all group activities. In 27% of the NHs, the residents did not have daily access to an outdoors area. The restrictions imposed by the epidemic prompted 75% of the NH to recruit extra staff. Furthermore, 45% depended on volunteers and 29% acquired personnel redeployed from institutions closed because of the lockdown. PPE Supply difficulties resulted in misuse of masks in 67% of NHs and of gowns in 25%.

###  Factors Associated With the Occurrence of at Least One Case of COVID-19 Among Residents

In multivariate analysis, the factors associated with decreased risk were in-room meal service, pre-emptive banning of visits, and daily access to a space outdoors. The total number of residents in the NH or per sector, the presence of double rooms, and human resources (presence of a physician, caregiver-to-resident ratio, total staff-to-resident ratio) had no influence on the risk of occurrence of a case. Misuse of PPE was also not associated with the occurrence of cases. The ORs of the explanatory variables included in the multivariate model are presented in Table 1.

**Table 1:** Odds Ratios and Significance of the Criteria Associated With the Occurrence in Nursing Homes of at Least One Case of COVID-19 Included in the Multivariate Analysis (Univariate *P* < .20)

	**Univariate Analysis**			**Multivariate Analysis**		
	**OR**	**95% CI**	***P***	**OR**	**95% CI**	***P***
Urbanization			.10			.13
City centre	1.00	-	-	1.00	-	-
Suburb	1.99	0.64-6.11	.23	4.38	0.85-26.16	.09
Isolated town	0.38	0.08-1.37	.16	0.54	0.06-3.78	.55
Rural area	0.70	0.22-2.14	.53	1.19	0.21-7.05	.85
Territorial subdivision			.09			.11
Finistère	1.00	-	-	1.00	-	-
Morbihan	1.20	0.21-6.76	.83	3.87	0.25-72.20	.33
Côtes d'Armor	1.59	0.28-9.02	.59	1.04	0.11-10.67	.96
Ille et Vilaine	3.65	1.13-16.31	.05	5.53	0.97-50.22	.08
Number of residents	1.00	1.00-1.01	.14	0.99	0.97-1.01	.36
Carers-to-resident ratio	0.01	0.00-2.82	.14	0.10	0.00-291.69	.60
Total staff-to-resident ratio	0.01	0.00-0.37	.02	0.34	0.00-140.21	.74
Residents-to-sector ratioa	1.12	1.05-1.21	<.01	1.07	0.93-1.26	.38
Hygienist			.03			.26
None	1.00	-	-	1.00	-	-
Intern to NH	2.23	0.76-6.65	.14	1.56	0.33-7.14	.57
Shared nurse from SCPNI	3.96	1.38-11.83	.01	3.88	0.76-21.96	.11
Ban on visits						
Early (before March 11, 2020)	1.00	-	-	1.00	-	-
When recommended or later	1.87	0.80-4.64	.16	5.27	1.29-27.63	**.03**
Mask wearing						
Early (before March 14, 2020)	1.00	-	-	1.00	-	-
From March 14, 2020	3.53	1.16-15.29	.05	3.38	0.71-22.72	.16
Residents confined to rooms						
No	1.00	-	-	1.00	-	-
Yes	0.29	0.11-0.69	<.01	0.36	0.08-1.54	.18
Daily access to an outdoor space						
No	1.00	-	-	1.00	-	-
Yes	0.42	0.18-1.04	.05	0.20	0.04-0.90	**.04**
In-room meal service for over 1 month						
No	1.00	-	-	1.00	-	-
Yes	0.11	0.04-0.28	<.001	0.10	0.02-0.35	**<.001**

Abbreviations: OR, odds ratio; CI, confidence interval; SCPNI, Support Centre for the Prevention of Nosocomial Infections; NH, nursing home; COVID-19, coronavirus disease 2019. ^a^Per 10 units.

###  Factors Associated With Overall Mortality

None of the observed factors was significantly associated with the mortality of residents in multivariate analysis. We found no effect of geographical location, pooled residents functional dependence score, available human resources, or the number of residents in the NH or by sector. Lockdown methods were not associated with the number of deaths. The ORs of the explanatory variables included in the multivariate model are presented in Table 2.

**Table 2:** Odds Ratios and Significance of the Criteria Associated With the Mortality Rate Included in the Multivariate Analysis (Univariate *P* < .20)

	**Univariate Analysis**			**Multivariate Analysis**		
	**OR**	**95% CI**	***P***	**OR**	**95% CI **	***P***
Territorial subdivision			.63			.58
Finistère	1.00	-	-	1.00	-	-
Morbihan	1.08	0.86-1.36	.48	1.07	0.85-1.34	.58
Côtes d'Armor	0.96	0.75-1.22	.75	0.89	0.69-1.15	.40
Ille et Vilaine	0.94	0.78-1.15	.55	0.94	0.76-1.15	.53
Pooled residents functional dependence score^a,b^	1.01	1.00-1.02	.10	1.01	1.00-1.02	.09
Residents-to-sector ratio^b^	1.01	1.00-1.02	.11	1.01	0.99-1.02	.32
In-room meal service						
No	1.00	-	-	1.00	-	-
Yes	0.86	0.73-1.02	.08	0.87	0.73-1.06	.16
Misuse of gowns						
No	1.00	-	-	1.00	-	-
Yes	1.17	0.97-1.39	.10	1.19	0.98-1.43	.07

Abbreviations: OR, odds ratio; CI, confidence interval. ^a^Evaluated with the French tool GIR used for NH funding. ^b^Per 10 units.

###  Resources Identified and Difficulties Reported

 Implementation of protective measures among professionals was deemed to be rather easy, except for social distancing. In contrast, it was harder to get residents to comply with the measures concerning confinement to rooms, hand hygiene, or social distancing (cf. Figure).

**Figure:**
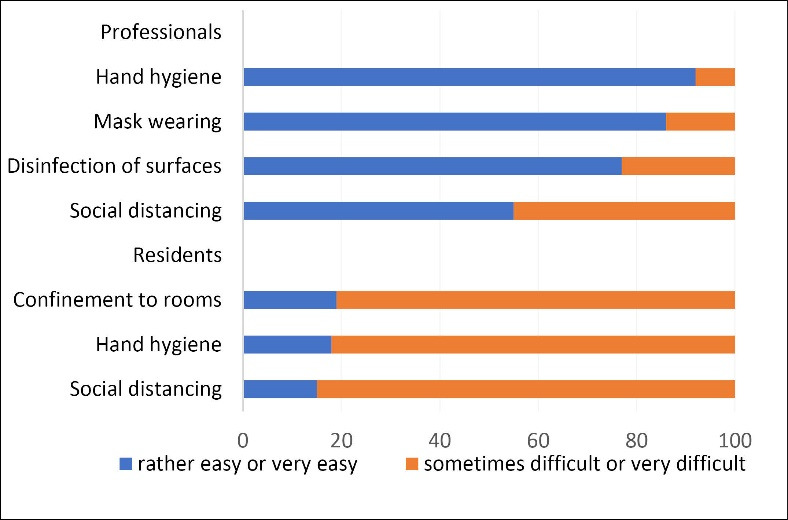
Difficulties Reported by Nursing Home Directors Concerning Observance of Protective Measures by Professionals and Residents.

 The main difficulty spontaneously mentioned were the lack PPE, including mainly masks but also gown, goggles, head and shoe cover, gloves. Hydroalcoholic solution or Disinfectant were also cited. Lack of human resources have been reported by 36% of respondents, whether medical and nursing, and social. Finally, the abundance of sometimes-contradictory recommendations (25% of respondents) appear as an additional challenge. Concerning useful resources, respondents bring forward all kind of formal or informal networks, and local solidarities. One fifthspontaneously mentioned redeployment of personnel from other institutions (closed hospital departments, schools, etc) who had become available because of lockdown-imposed cessation of activities. Concerning access to and sorting of information, 58% of respondents mentioned as sources the support services (regional support centre for quality of care, geriatric hotline, and Support Centre for the Prevention of Nosocomial Infections).

## Discussion

###  Identified Risk Factors

In-room meal service, daily access to outdoor spaces, and early ban on visits were associated in our study with reduced outbreak risk. The central role of communal dining in transmission has already been pointed out in studies conducted in the general population.^28^ Nonetheless, to our knowledge this is the first time it has been shown in aged-care settings. This result should be weighed against the fact that communal dining is associated with an increase in energy intake.^29^

 Room lockdown was implemented by nearly two-thirds of NHs, at the risk of a large impact on residents’ quality of life. In one-quarter of NHs the residents did not even have daily access to an outdoors space. Our results indicate that room lockdown, observance of which by residents with dementia syndrome is difficult to enforce, does not result in better control of the risk of outbreak than cessation of communal dining.

 The effect of an early ban on visits should be interpreted in a context where visits took place without the wearing of PPE. It is therefore hard to extrapolate to other situations and bans on visits cannot be recommended for a prolonged period for obvious ethical reasons.

The third factor associated with decreased risk of an outbreak in an NH is more surprising: daily access to an outdoors area for all asymptomatic residents. While it is well known that the transmission of severe acute respiratory syndrome coronavirus 2 (SARS-CoV-2) is more frequent indoors than outdoors,^30^ this association was unexpected. This result can be compared to that of the case-control ComCor study,^31^ which found an association between outdoor physical activity and reduced risk of SARS-CoV-2 infection. In the absence of a known pathophysiological hypothesis explaining this association, there may be confounding factors not taken into account by our questionnaire relating to NH architecture and garden layout,^32^ or to the quality of management.

Unlike the results reported by White et al in the United States,^33^ we found no effect of the total number of residents on the occurrence of outbreaks in NHs. This may be linked with better operational support (management, hygiene, etc) in the largest NHs, or with widespread room lockdown, which minimized the impact of the total number of NH residents. Rolland et al^34^ found that compartmentalization of staff, and not of residents, was associated with reduced occurrence of outbreaks, the risk being analyzed for professionals and residents combined.

Observed mortality correspond to what was expected in comparison with mortality rate in long term care facilities in 2018 and 2019 spring in France (2% par month).^35^ We identified impact of lockdown measures on confirmed epidemic occurrence but not on total mortality. This leads us to exclude the hypothesis that NH epidemics could have occur in a significant number NH without any positive testing. On the other hand, this should not overshadow the potential adverse consequences of lockdown in terms of loss of autonomy, malnutrition and mental suffering,^27^ which may alter the quality of and, in time, the length of life. The lack of relation between the rigour of lockdown and overall mortality leads us to exclude the hypothesis of a significant number of NHs affected by outbreaks that were unconfirmed because testing was not done.

###  Low Incidence Rate and Strict Lockdown Measures

Our results give a precise view of the consequences of the COVID-19 epidemic for NH residents in Brittany, in terms of morbidity-mortality, but also living conditions.They are consistent with official data of COVID-19-related deaths in NH in Britany during this period (110 death^24^ in all regional NH, 64 in our survey with a participation rate of 47%). However, the ratio of NH with at least one case differs in our sample (10%) from the ratio calculated from official figures (70/494=14%). This raises concerns on possible response bias, with more replies from NH with lower infection rates

According these official data, NH residents accounted for 34% of deaths in Brittany (110/326). This confirms that NHs in Brittany were relatively spared by the epidemic, with 10% to 14% of them affected during this period, compared with approximately 40% of NHs in France as a whole.^23^

Official figures report 110 COVID-19-related deaths in NHs and 326 deaths in total in Brittany during this first wave,^24^ thus indicating that NH residents accounted for 34% of deaths. As a comparison, this proportion during the first wave was around 47% for France as a whole, 41% in the United Kingdom, 78% in Canada, and 42% in the United States.^25^ In our study, the attack rate remained low after the first cases in most NHs (average attack rate 9%), compared with an attack rate of 45% in a recent meta-analysis.^26^ Even if asymptomatic cases have not been taken in account, this rate seems low, and is probably linked with the low rate in general population.^36^ Our study was not designed to explain this low rate. Mortality rate is higher in our survey than in other studies in NH during the same period (26% versus 11% in Burgaña Agoües et al^37^), which is logical as we considered only symptomatic cases.

 The early and strict implementation of lockdown measures may seem a posteriori surprising given the low impact of the epidemic in Brittany (6% of deaths in NHs attributed to COVID-19). In a context of great uncertainty and anxiety, directors often preferred to anticipate and then go beyond already strict governmental recommendations, notably concerning the possibility of confining residents to their rooms.

In NHs, no increase in mortality rate was associated with the severity of the lockdown measures. This should not lead to hasty reassurance, given the potential consequences of lockdown in terms of postural maladaptation, malnutrition and mental suffering,^27^ which may alter the quality of and, in time, the length of life. Our observations also raise numerous ethical questions concerning respect of the rights and of the well-being of NH residents.

###  Strength and Limitations

The strength of our study lies in its scale. The 231 NHs that completed our questionnaire appear to be representative of NHs in Brittany, in terms of status,^7^ size, and the average degree of loss of independence of the residents.^38^ The number of probable cases should therefore be interpreted with caution. *That is why we choose as primary outcome the occurrence of at least one confirmed case (possible only if two similar symptomatic cases were present), even if some of the studied factors may impact the spread of the epidemic and not its introduction*. Interpretation of our results must take into account the context of this first wave of the epidemic, with a generalized shortage of PPE,^22^ and the fact that Brittany was finally little affected during the first wave of the epidemic, because of early lockdown of the whole population.^2^

## Conclusion and Implications

Our results show that control of COVID-19 outbreaks in NHs is possible, even in the absence of available vaccine and when PPE supply is difficult, if the spread of the epidemic that remained limited in the community. Because of the presymptomatic transmission of COVID-19,^39^ the control involved the implementation of strict preventive measures prior to the diagnosis of the first case.

 This good epidemiological result should not tempt us to minimize the effects of restricting two-thirds of residents to their rooms. These effects could not be studied prospectively, because of the context of the crisis and also of lockdown itself, which prevents any outside view of residents’ living conditions. Beyond potential adverse physical and psychological consequences for residents, it raises ethical questions concerning respect of their rights.

 Our results can enlighten decision makers faced with a risk of outbreaks in NHs. Cessation of communal dining seems to be the main measure likely to be effective in preventive terms. It does not seem that room lockdown and cessation of group activities should be recommended, particularly if mask wearing is possible. It is desirable that residents should have access to outdoor areas.

## Acknowledgements

 The authors thank the CPias (Centre d’appui pour la Prévention des Infections liées aux Soins), Dr. Gael Durel of the Association des Médecins Coordonnateurs, and the NH directors Michel Barbe, Damien Visseaux and Ilda Ferreira who agreed to test the questionnaire.

## Ethical issues

 No personal data concerning residents were collected. Data were processed in accordance with reference methodology and reported to the French Data Protection Authority (CNIL). The institutional review board approved this study (Opinion 20.89).

## Competing interests

 Authors declare that they have no competing interests.

## Authors’ contributions

 Study concept and design: AC, KC, JFV, and DS. Acquisition of data: AC. Analysis and interpretation of data: AC, KC, JP, FL, FO, JFV, and DS. Drafting of the manuscript: AC, KC, JP, JFV, and DS. Critical revision of the manuscript for important intellectual content: AC, KC, JP, FL, FO, JFV, and DS.

## Funding

 This work was supported by the French Ministry of Solidarity and Health.
